# The Repair of Graves and Head-Stones

**Published:** 1921-04-09

**Authors:** 


### The Repair of Graves and Head-Stones.

### A DIFFICULT PROBLEM UNDER WILLS.

BEQUESTS to institutions are often conditioned by the testator's wish that family vaults and tombstones shall be kept in good order. An incorporated hospital with perpetual succession has been invited to undertake such a responsibility, but the testator declares his wish not to impose or create a trust. The board of management desires to carry out the testator's wishes, but is not sure how best to undertake the responsibility. It has recorded acceptance on the minutes, but recognises that the machinery for carrying out the work now and in the future is not existent. Keen interest is taken by many people in family vaults and graves, and considerable time and money are spent in their supervision and repairs. Wishes expressed by testators must, if accepted at all, be accepted in full sincerity and intention.

The decision, however, of a board to-day is not necessarily binding on a board of the future, and the question, therefore, arises how the acceptance of the responsibility can best be handed on to future administrators.

A record of such bequests might be inserted in the annual report, but the record is not permanent because circumstances sometimes restrict the report to a few pages. During the war, for example, only the essential accounts were given by many hospitals. A record might be engrossed on vellum or parchment, framed, and placed in the board-room. Both means help to indicate the responsibility which has been accepted, but even then the means by which graves can be inspected from time to time and their condition reported on is not indicated.

The matter is important, and the columns of THE HOSPITAL are open to hospital administrators to suggest the best means for giving effect to testamentary bequests of the kind. It is, of course, understood that the bequests referred to are bequests which do not create permanent trusts. It has, we believe, been held by the Courts that a bequest creating a permanent trust for the upkeep or repair of gravestones or graveyards fails in so far as the attempt to create a trust is concerned, although the charitable bequest does not fail.

All institutions are interested in the question, and we hope the opportunity to put forward suggestions will be taken advantage of.

